# Exploring the Surge in Paediatric Type 2 Diabetes in an Inner-City London Centre—A Decade-Long Analysis of Incidence, Outcomes, and Transition

**DOI:** 10.3390/children11020173

**Published:** 2024-01-29

**Authors:** Farah Abdelhameed, Anna Giuffrida, Ben Thorp, Myuri K. Moorthy, Evelien F. Gevers

**Affiliations:** 1 Barts Health NHS Trust—Royal London Children’s Hospital, London E1 1BB, UK; farahfahdmahmoud.abdelhameed@uhcw.nhs.uk (F.A.); giuffrida.anna@studium.unict.it (A.G.); ben.thorp1@nhs.net (B.T.); myurikrishna.moorthy@nhs.net (M.K.M.); 2 William Harvey Research Institute, Barts and The London Medical School, Queen Mary University of London, London EC1M 6BQ, UK; 3 School of Medicine, University of Catania, 95124 Catania, Italy

**Keywords:** paediatric type 2 diabetes, complications, single-centre cohort, youth, obesity

## Abstract

The rising prevalence of paediatric type 2 diabetes (T2D) is concerning, particularly with limited medical intervention despite evidence of accelerated disease progression. This study of a Barts Health NHS Trust cohort from 2008 to 2022 aims to elucidate the incidence, clinical outcomes, and complications associated with paediatric T2D. A retrospective analysis utilising electronic and paper records identified 40 patients with T2D. The incidence doubled from 2.6/year in 2008–2013 to 5.4/year in 2014–2018. Sixty-eight percent exhibited co-morbidities, notably learning disabilities. At diagnosis, the mean BMI was 32.4 ± 6.71 kg/m^2^, with no gender-based disparity and no significant change over a two-year follow-up. The initial HbA1c was 75.2 ± 21.0 mmol/mol, decreasing to 55.0 ± 17.4 mmol/mol after three months (*p* = 0.001) and then rising to 63.0 ± 25.5 mmol/mol at one year (*p* = 0.07). While 22/37 patients achieved HbA1c < 48 mmol/mol, only 9 maintained this for a year. Several metabolic and cardiovascular complications were observed at diagnosis and follow-up, with no significant change in frequency. In 2022, 15 patients transitioned to adult services. HbA1c at transition was 74.7 ± 27.6 mmol/mol, showing no change one year post-transition (71.9 ± 26.9 mmol/mol, *p* = 0.34). This study highlights substantial therapeutic failure, with current management falling short in achieving a sustained reduction in BMI or HbA1c. Novel treatment approaches are needed to improve clinical outcomes and address the high burden of co-morbidities and complications.

## 1. Introduction

In the UK, 1 in 16 people are estimated to have diabetes, with type 2 diabetes (T2D) accounting for 90% of all cases [1,2]. Although typically considered a disease of adulthood, T2D has been increasingly recognised in children and adolescents over the last two decades [3]. The SEARCH study reported an increasing incidence by 4–5% per year in the USA, mirroring the paediatric obesity epidemic [4,5]. The Royal College of Paediatrics Spotlight Audit for T2D in children in England also suggested that the incidence has increased from 0.7/100,000 in 2015 to 1.7/100,000 in 2019 [6,7]. Currently, limited data exist on how the demographics, presentation, and outcomes of T2D in children and adolescents have changed over the past decade in the UK and in local areas [1,8].

Risk factors for T2D include ethnicity, obesity, female gender, and a family history of diabetes [9]. Several studies across the UK and globally have demonstrated that certain ethnicities such as Asian and Afro-Caribbean are more susceptible to developing T2D due to genetic predispositions [7,10,11]. T2D also tends to affect youth from lower socioeconomic status disproportionately, paralleling the disparities seen in the incidence of obesity [9,12,13].

Children and adolescents with T2D have a more aggressive disease evolution compared to adults [14,15,16,17]. They have a greater risk of micro- and macro-vascular complications compared to patients diagnosed with T2D at an older age, but also compared to patients with T1D [18,19,20]. The TODAY trial showed that amongst 704 youths diagnosed with T2D, 80% had low HDL cholesterol, 26% had hypertension, and 10% had hypertriglyceridemia at diagnosis [21]. Studies have also demonstrated that microvascular complications in youth-onset T2D can manifest within 5 years of diagnosis, whilst deterioration in glycaemic control can begin within 2 years after diagnosis [22,23].

Diabetes complications can be reduced by effective diabetes management with early detection of complications [24]. The aim of treatment is to achieve normoglycaemia and to adequately manage co-morbidities and complications to prevent future deterioration [18,24]. Lifestyle modifications, namely diet and physical activity, are typically considered first-line treatment [25]. However, whilst lifestyle intervention has been shown to be effective in adult-onset T2D, this remains to be established in youth-onset T2D [18]. The TODAY study is the only trial to date that has investigated the value of lifestyle intervention on a large scale, concluding that lifestyle modifications with metformin was not superior to metformin alone in maintaining glycaemic control in adolescents with T2D [26]. Additionally, there was no improvement in cardiovascular risk factors incurred by lifestyle modification when combined with metformin [26].

Overall, the rising prevalence of youth-onset T2D is of growing concern due to the significant clinical and economic burden it poses. Medical treatment of T2D in children and adolescents is significantly restricted due to the lack of evidence compared to adults, despite the suggestion of a more aggressive disease progression. The objective of our study is to analyse the presentation, management and outcomes of children and adolescents diagnosed with T2D over the last 10–15 years in our local area in London, in order to inform future care strategies.

## 2. Materials and Methods

We conducted a retrospective cohort study at Barts Health NHS Trust—Royal London Hospital, which is one of the larger paediatric diabetes centres in England and Wales. Patients under the care of the Paediatric Diabetes Team at the Royal London Children’s Hospital between 1 January 2008 and 31 December 2018 were selected from the electronic database Twinkle and data were collected for this time period. Additional data were collected in September 2022 of patients who had transitioned to adult care services from the original cohort identified.

Fifty-five patients coded with a diagnosis of T2D were identified during the selected time period and further reviewed to confirm the diagnosis. T2D was defined according to the International Society of Paediatric and Adolescent Diabetes (ISPAD) 2018 criteria: two-hour post-load glucose ≥ 11.1 mmol/L during an oral glucose tolerance test or HbA1c ≥ 48 mmol/mol or fasting plasma glucose ≥ 7.0 mmol/L along with evidence of reduced insulin sensitivity and no clinical evidence of T1D [27]. Fifteen patients had no or insufficient clinical data available either electronically or on paper notes and were excluded from the analysis. Therefore, the overall sample included 40 patients. All patients in the UK and in our cohort have access to free treatment under the National Health Service and do not need to pay for drugs or for prescriptions if less than 18 years of age or in full-time education.

### 2.1. Data Collection

A review of the electronic records and paper notes was performed to collect patient data. Data collection was until transfer to adult services or until no more data were available either because the patient stopped attending or due to the patient relocating within the study time period selected.

Demographic characteristics—Age at diagnosis, gender, age at transition (if applicable) and ethnicity were collected. Ethnicity was reported according to the most specific description, and where one ethnicity could not be determined, the patient was categorised as ‘Other’. The Income Deprivation affecting Children Index (IDACI) was calculated using public data (https://imd-by-postcode.opendatacommunities.org/imd/2019, accessed on 13 January 2024).

Clinical parameters—Clinical parameters at diagnosis, 3 months, 6 months, 9 months, 1 year, 2 years, and 3+ years post-diagnosis (±10% of time window) were collected. Body mass index (BMI) was recorded, and BMI standard deviation score (BMI SDS) was calculated using UK-Cole data [28]. HbA1c and random blood glucose data were also collected. Antibody titres for anti-GAD (glutamic acid decarboxylase), islet cell, tyrosine phosphatase-related islet antigen 2, insulin IgG, and anti-ZnTF8 were collected since 2014. Co-morbidities were recorded on the basis of a formal diagnosis at the time of T2D diagnosis. Genetic investigation was performed if there were concerns of underlying genetic syndromes; however, whole-genome sequencing was not available at the time of diagnosis for these patients. One patient was known to have PWS; no other genetic syndromes were identified. MODY testing was performed when clinically indicated with criteria of the Exeter genetics lab. No MODY was identified in the cohort.

Treatment data—Data on metformin, types and doses of insulin or other anti-hyperglycaemic drug treatment was collected at start of treatment and during follow-up.

Complication outcomes—Data on hypertension, raised alanine transaminase (ALT), sleep apnoea, fatty liver disease on imaging, and dyslipidaemia were collected at diagnosis and follow-up. Hypertension was defined as 2 independent blood pressure readings above the 95th percentile adjusted for age, gender and height using the 4th Taskforce report data on paediatric and adolescent hypertension [29]. Raised ALT was defined as more than twice the upper limit of normal. Microalbuminuria was assessed using the urine albumin/creatinine ratio (UACR) > 3 mg/mmol according to ISPAD guidelines [27]. Sleep apnoea was determined on either clinical suspicion or a formal diagnosis in the patients’ medical history, whilst fatty liver disease was only recorded based on radiological ascertainment. Dyslipidaemia was determined according to ISPAD guideline cut-offs of low-density lipoproteins (LDL) levels > 2.6 mmol/L and cholesterol > 5 mmol/L [27]. Blood pressure, ALT, LDL, and cholesterol levels were also recorded at the same follow-up time points as mentioned above.

### 2.2. Statistical Analyses

All continuous demographic and clinical variables were expressed as mean and standard deviation, whilst qualitative variables were presented as frequencies in percentages. Student *t*-test was used for normally distributed data and a Mann–Whitney U test was used for non-normally distributed data. Chi-squared test was used for nominal variables to compare groups. SPSS version 25 was used, and a *p* ≤ 0.05 was considered to be statistically significant.

## 3. Results

### 3.1. Participant Characteristics at Diagnosis

**Figure 1:**
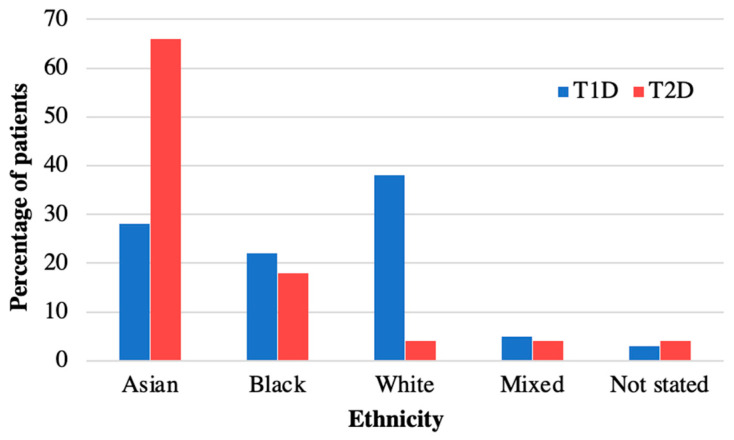
**Ethnicity of patients with T2D versus T1D in diabetes cohort at Royal London Children’s Hospital.** Ethnicity of patients with T2D in 2008–2018 compared to T1D. Patients with T2D are more often from Asian ethnicity than in T1D.

Table 1 summarises the characteristics of the 40 patients diagnosed with T2D during the time period 2008–2018 and included in the analysis. The mean age at diagnosis was 13.9 (SD 1.73) years, median 14.1 years, and similar for both sexes (Table 1). There was a female predominance (63%, *n* = 25) and 60% had South-East Asian ethnicity (Table 1). The distribution of ethnicities was compared to our cohort of patients with type 1 diabetes (T1D) (*n* = 350) from the same demographic area, and this confirmed a higher prevalence of T2D in South-East Asians compared to T1D (Figure 1). The postcode linked Income Deprivation affecting Children Index (IDACI) measures the proportion of 0–15-year-olds living in low-income households ranking from 0 (low) to 1 (high). Our group of patients with T2D had a significantly higher mean score than our patients with T1D (0.46 (0.02) vs. 0.38 (0.15), *p* = 0.02). The number of newly diagnosed T2D patients per year increased from 2.6 patients/year during 2008–2013 to 5.4 patients/year during 2014–2018.

#### 3.1.1. Clinical Parameters

##### Co-Morbidities and Antibody Status

**Figure 2:**
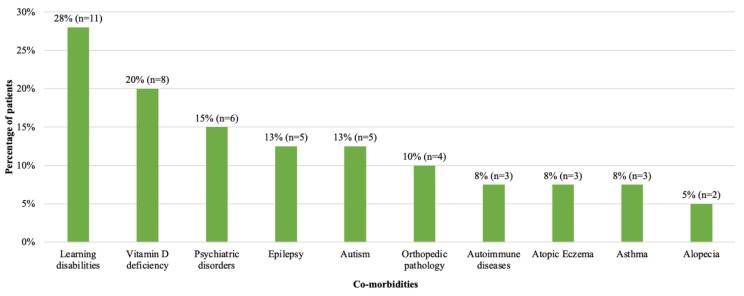
**Frequency of co-morbidities at diagnosis in patients with T2D diagnosed in 2008–2018**. Number and percentage of patients with co-morbidities are shown. Learning disability, vitamin D deficiency and psychiatric disorders were the most common co-morbidities. One patient with learning disability had a diagnosis of Prader Willi Syndrome.

Sixty eight percent of patients (*n* = 27) had additional conditions at diagnosis of T2D (Figure 2). Learning disabilities and vitamin D deficiency were the most common. Vitamin D deficiency (*n* = 8) was more common in females (88%, *n* = 7/8) and learning disabilities were more common in males (47%, *n* = 7/15) than in females (16%, *n* = 4/25). Six patients (15%) had mental health disorders; more specifically, one patient each had anxiety disorder, depression, dissociative personality disorder, Tourette syndrome, obsessive compulsive disorder and conduct disorder. Two patients had positive autoantibodies (both anti-GAD) but were still deemed to have T2D on the basis of their clinical presentation including a high C peptide level (Patient 1: C-peptide 2572 pmol/L, Patient 2: C-peptide 6200 pmol/L). Patient 1 was also able to achieve a reduction in their HbA1c to 44 mmol/mol with metformin only. At the time these patients were diagnosed, there was no requirement to have negative antibodies for a diagnosis of T2D using ISPAD guidelines, in contrast to the latest ISPAD 2022 guidelines [27]. We therefore included these two patients in the analysis. Of note, these patients have not developed Type 1 diabetes since 2018.

##### BMI and BMI SDS

BMI at diagnosis was 32.4 (SD 6.71) kg/m^2^, with a mean BMI SDS of 2.87 (SD 0.70) (Table 1). Table 2 shows the clinical parameters for males and females. BMI and BMI SDS at diagnosis were not statistically different between sexes (Table 2). Clinical parameters were compared between the two time periods of diagnosis (Table 3). Patients diagnosed during 2014–2018 had a higher BMI SDS at diagnosis, compared to patients diagnosed during 2008–2013 (*p* = 0.02) (Table 3).

##### HbA1c

Mean HbA1c at diagnosis was 75.2 (SD 21.0) mmol/mol with a mean random blood glucose of 10.6 (SD 4.14) mmol/L (Table 1), with no difference between sexes (Table 2). In 2008–2013 mean HbA1c was higher at diagnosis compared to 2014–2018 (89.0 (SD 19.2) mmol/mol vs. 69.4 (SD 19.2) mmol/mol, *p* = 0.02) (Table 3).

##### Complications

Assessment of complications of obesity and T2D at diagnosis was incomplete amongst our cohort. A total of 53% of patients were assessed for hypertension, 75% for raised ALT, 35% for microalbuminuria, 65% for sleep apnoea, 60% for fatty liver on ultrasound, and 75% for dyslipidaemia. In those that were assessed, the complication rate was high at diagnosis (Table 4). Only 18% (*n* = 7) of patients were assessed for all five complications, and one patient had all five complications. Hypertension (43%) and dyslipidaemia (high LDL) (50%) were the most frequent complications (Table 4). Three patients (8%) had no complications at diagnosis.

##### Treatment

The treatment regimen for the patients is summarised in Supplementary Table S1. Metformin was prescribed at diagnosis for 95% of patients with an average dose of 895 (SD 371) mg/day. In addition, long-acting insulin (mean dose 0.30 (SD 0.16) U/kg) and short-acting insulin (mean dose 0.42 (SD 0.20) U/kg) were prescribed at diagnosis to 38% (*n* = 14/37) and 33% (*n* = 6/18) of patients. During follow up, the mean dose of short acting insulin remained similar, but there was a trend of higher doses of long-acting insulin and total insulin by approximately 23% by the third year of follow-up.

### 3.2. Clinical Progression during Treatment Follow-Up

#### 3.2.1. Clinical Parameters

##### BMI and BMI SDS

BMI for the cohort remained the same over three years of follow-up, 32.4 (SD 6.71) kg/m^2^ at diagnosis to 33.9 (SD 6.02) kg/m^2^ after 3 years, without a difference between sexes (Table 2). Patients diagnosed in 2008–2013 had a mean BMI of 32.0 (SD 4.61) kg/m^2^ and 31.5 (SD 5.34) kg/m^2^ at the first and second year of follow-up, respectively, which was not significantly different from diagnosis (Table 3). Likewise, patients diagnosed in 2014–2018 had no statistically significant changes in mean BMI during follow-up (first year: 33.4 (SD 7.30) kg/m^2^, second year: 34.0 (SD 7.49) kg/m^2^).

**Figure 3:**
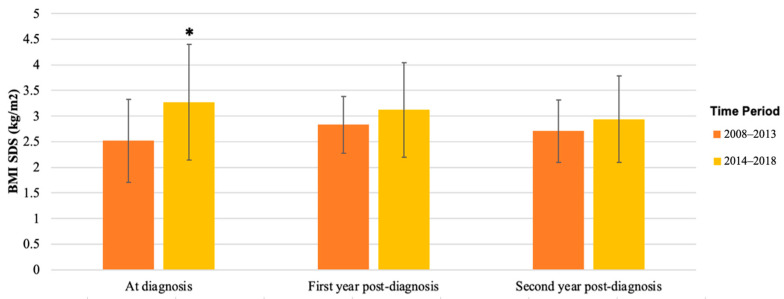
**Mean BMI SDS of patients with T2D diagnosed in 2008–2013 versus 2014–2018.** BMI SDS at diagnosis, first year post-diagnosis, and second year post-diagnosis in patients was collected from patient records; mean and SD are shown. Mean BMI SDS was significantly higher in patients diagnosed in 2014–2018 at diagnosis (* *p* = 0.02), but in the first and second year after diagnosis, there was no significant difference between the groups.

BMI SDS also remained similar during follow-up, 2.87 (SD 0.70) at diagnosis and 2.98 (SD 0.69) after 3 years. Despite the significant difference in BMI SDS between the two time periods at diagnosis (*p* = 0.02, Table 3), BMI SDS became similar during follow-up (Figure 3). Supplementary Figure S1 shows the individual tracking of BMI SDS for each patient for whom data were available at diagnosis and during follow-up.

##### HbA1c

**Figure 4:**
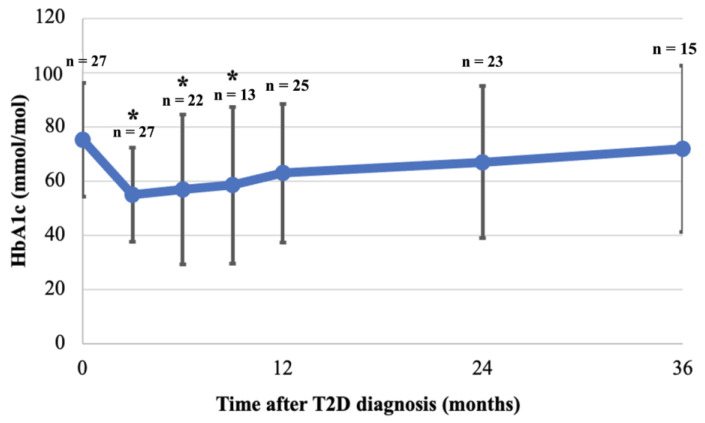
**Mean HbA1c at diagnosis and during 36 months follow-up.** HbA1c was collected for patients at 3, 6, 9, 12, 24 and 36 months (see methods). Mean and SD are shown, as are the number of patients with available HbA1c at each time point (n). Mean of available HbA1c at each time point was compared to mean HbA1c at diagnosis; HbA1c was significantly lower at 3, 6, 9 months after diagnosis but not thereafter (* *p* < 0.05).

The mean HbA1c at diagnosis was 75.2 (SD 21.0) mmol/mol and decreased significantly during the first 9 months of follow-up with a nadir at 3 months (55.0 (SD17.4) mmol/mol, *p* = 0.001), 6 months (56.9 (SD 27.5) mmol/mol, *p* = 0.01), and 9 months (58.5 (SD 28.8) mmol/mol, *p* = 0.04) (Figure 4). At 12 months follow-up, mean HbA1c was not significantly lower anymore compared to diagnosis (63.0 (SD 25.5) mmol/mol, *p* = 0.07), and continued to rise slowly towards baseline HbA1c by the third year of follow-up (Figure 4). HbA1c in females at one year follow-up was significantly lower than at diagnosis (diagnosis: 77.4 (SD 22.0) mmol/mol, first year: 60.9 (SD 21.9) mmol/mol, *p* = 0.03, but increased again in the second year. However, this was not seen in HbA1c for males (diagnosis: 69.9 (SD 18.4) mmol/mol, first year: 66.7 (SD 32.0) mmol/mol, *p* = 0.80). No statistical differences were found between males and females during follow-up (Table 5). Despite the difference in mean HbA1c between patients diagnosed in 2008–2013 and in 2014–2018 at diagnosis, the mean HbA1c was not statistically different between the two groups during follow-up (Table 5).

##### Complications

The frequency of complications remained high during follow-up (Table 4). Hypertension and dyslipidaemia were still the most common, although a lower proportion had hypertension compared to diagnosis (35% vs. 43%). Microalbuminuria was noted in more than double the patients found at diagnosis (33% vs. 14%), whilst fatty liver disease was found in only 19% of patients during follow-up compared to 29% at diagnosis (Table 4). No significant differences were noted in the frequencies of complications between diagnosis and follow-up.

##### Treatment and HbA1c Outcomes

The maximum dose for metformin was near 2000 mg per day but seven patients reduced their dosage and a further six patients stopped metformin due to side effects (Supplementary Table S1). The percentage of patients on insulin treatment increased by 48% over the 3 years of follow-up (diagnosis: 56% (*n* = 14/25), third year: 83% (*n* = 10/12)), but mean doses of both long- and short-acting insulin did not change (Supplementary Figure S2). Fifty nine percent (*n* = 22/37) achieved an HbA1c < 48 mmol/L at least once during follow-up. Nine patients (*n* = 9/37, 24%) achieved an HbA1c < 48 mmol/mol for more than a year, with two of those patients relapsing and three patients remaining on insulin. Seven of those nine patients were able to maintain their HbA1c < 48 mmol/mol for more than two years.

### 3.3. Clinical Outcomes following Transition to Adult Care Services

Thirty patients had transitioned to the adult diabetes services by September 2022. Of these, seven patients were lost to follow-up or discharged to their GP due to non-attendance and four patients were discharged to their GP due to diabetes reversal. We were able to obtain the records of 15 patients that still remained in the adult services. The mean age at transition was 17.7 (SD 1.35) years, with an average duration after diagnosis of 57.6 (SD 13.8) months. HbA1c one year after transition was available for nine patients. Supplementary Figure S3 shows the individual tracking of HbA1c during and after transition for these nine patients. Mean HbA1c one year after transition was 71.9 (SD 26.9) mmol/mol compared to 74.7 (SD 27.6) mmol/mol at transition (paired *t*-test: *p* = 0.34) and 74.7 (SD 24.9) mmol/mol at diagnosis (paired *t*-test, *p* = 0.81) for these patients. Two patients achieved an HbA1c < 48 mmol/mol one year after transition, of which one maintained an HbA1c < 48 remaining only on metformin. Prior to transition, no patients were on SGLT2 inhibitors or GLP1 agonists as these were not licensed yet for people < 18 years of age in the UK. The treatment regimen for the fifteen patients in the adult services consisted of metformin for most patients, combined with insulin treatment (*n* = 3), other anti-hyperglycaemic agents (*n* = 4), a combination of insulin and anti-hyperglycaemic agents (*n* = 4), metformin alone (*n* = 3) and one patient was on diet control only. The anti-hyperglycaemic agents included oral medication (*n* = 6) or combination of both oral and injectable drugs (*n* = 2). In terms of complications, 22% (*n* = 2/9) of patients had high cholesterol and 50% (*n* = 2/4) had an abnormal ALT, with a further 22% (*n* = 2/9) having hypertension in the first year of transition.

## 4. Discussion

The shifting landscape of diabetes in the UK reveals a departure from its traditional occurrence in adulthood, and it is now affecting a younger demographic, predominantly females from specific ethnic minorities and from areas with high deprivation. In our study, spanning 2008–2018 within a single trust, we evaluated the presentation, treatment, and outcomes of a young T2D cohort. This aimed to assess the effectiveness of current management strategies on disease progression, laying the groundwork for future considerations and adjustments to the care provided.

### 4.1. Epidemiology

The incidence of paediatric T2D in our cohort doubled between 2008–2013 and 2014–2018. Incidence data in Europe are limited, but our findings mirror the rising youth T2D rates worldwide [3,30]. An association between heightened T2D prevalence in socioeconomically disadvantaged areas, observed in our cohort, corresponds with national reports as outlined in the Royal College of Paediatrics Spotlight Audit for T2D in children and trends identified amongst the UK adult population [6,31]. The Spotlight Audit assessed all patients with T2D under the care of a paediatric diabetes team in England and Wales in 2019–2020 and showed that children and young people with T2D were more often from ethnic minorities and that 45.2% lived in the most deprived areas as compared to 23.2% for T1D [6]. The age range of presentation occurs concomitantly with puberty, a period marked by a transient reduction in insulin sensitivity [32,33]. High-risk ethnic groups such as South Asians are overrepresented in our T2D cohort compared to T1D as they are predisposed to obesity and cardiovascular disease due to genetic and environmental factors [30,34]. A UK national surveillance unit supports this trend, reporting elevated T2D incidences amongst children of ethnic minorities such as Asians and Afro-Caribbeans compared to white ethnicity [7]. T2D susceptibility involves genetic and environmental factors, with shared familial habits playing a key role [34,35]. Comprehensive management strategies should consider family history, encompassing cultural practices, to address the holistic context of the condition.

Our study identifies a notable prevalence of learning disabilities, particularly among males, a previously unreported observation to our knowledge. Plausible explanations include limited mobility, reduced exercise, or suboptimal dietary habits in affected children. Additionally, some of these patients may have undiagnosed syndromes predisposing them to diabetes or obesity, or that increased scrutiny of patients in the healthcare system may contribute to more thorough T2D assessments. As genetic knowledge advances, there is merit in considering whole-genome sequencing for T2D patients with learning difficulties, especially in the absence of acanthosis nigricans. The elevated co-occurrence of epilepsy and autism in this cohort, along with prevalent vitamin D deficiency, underscores distinctive characteristics warranting attention in T2D management [36,37]. 

### 4.2. Clinical Parameters

The mean BMI at diagnosis in our cohort (32.4 kg/m^2^) aligns with previous reports in European youth with T2D [38]. Contrasting studies indicate higher BMIs (35–39 kg/m^2^) at T2D onset in youth, which may potentially be attributed to geographical, ethnic, and age-related variations [39]. Initial management for T2D involves lifestyle and dietary interventions; however, their limited efficacy in youth is acknowledged [40]. Notably, in the TODAY study, adding intensive lifestyle intervention to metformin showed no significant weight loss versus metformin alone [26]. Our cohort demonstrated the declining efficacy of lifestyle intervention over three years, with a reduction of 0.2 kg/m^2^ or more in BMI SDS amongst 28% of patients by the first year compared to only 15% by the third year of follow-up. This suggests the complexity of inducing clinically significant BMI changes in youth compared to adults for whom lifestyle changes are more successful. Effective interventions for youth-onset T2D may require a multifactorial approach beyond diet and physical activity, incorporating cultural sensitivity given the higher prevalence amongst ethnic minorities and a family-based strategy for improved adherence [9].

In contrast to BMI, HbA1c initially decreased during the first year of treatment, with 76% of patients achieving a reduction exceeding 0.2%. However, by the end of the first year, HbA1c increased, nearly reverting to baseline levels by the third year; a trend observed in other cohorts [23,41]. The fluctuation may stem from a combination of adherence to medical and lifestyle interventions and declining beta-cell function [42,43]. The TODAY trial demonstrated that despite rigorous medical attention, nearly half of participants failed to attain adequate glycaemic control, irrespective of their intervention arm, suggesting a more intricate disease pathophysiology perhaps more aggressive than in adults [26]. In our cohort, females initially reduced their HbA1c significantly, although this was not sustained during subsequent follow-up, with the reason for this sex-based difference remaining unclear.

HbA1c and BMI SDS demonstrated a correlation with the decade of diagnosis. Patients diagnosed between 2008 and 2013 exhibited higher HbA1c but lower baseline BMI SDS compared to those diagnosed between 2014 and 2018. The elevated HbA1c during 2008–2013 may be attributed to T2D’s novelty in children, potentially leading to delayed diagnoses, unlike the more frequent screenings in 2014–2018. Despite a lower HbA1c at diagnosis in the latter group, no sustained difference in HbA1c during follow-up was observed. This implies that any potential advantage of earlier detection did not persist over time, suggesting a need for more effective management strategies. The recent introduction of liraglutide and anticipated outcomes from other drug trials offer promising avenues for enhancing paediatric T2D patients’ access to wider medical treatments [44].

Our analysis of the transition data found that around 25% of our patients were lost to follow-up during the transition to adult care, similar to the findings of the SEARCH study [45]. In contrast to SEARCH, which demonstrated worsening of HbA1c during transition, our study revealed no significant changes in HbA1c during transition or one-year post-transition compared to diagnosis in patients that were successfully transitioned to adult care. Despite the availability of wider range of drug options for adults (>18 years) with T2D, our cohort showed no significant HbA1c reduction. This emphasises the necessity for tailored transition services for young adults with youth-onset T2D, who may require different management strategies from adults diagnosed after 18 in order to optimise their outcomes.

### 4.3. Complications

Assessment of diabetes complications at diagnosis was frequently incomplete in our study, mirroring the Spotlight Audit results in England and Wales [6]. Youth-onset T2D is associated with a high frequency of complications at diagnosis, evident in our cohort and consistent with studies highlighting an accelerated development of complications compared to adults with T2D or children with T1D [46,47,48]. Our finding that fatty liver disease was exclusive to non-white ethnicities aligns with reports of a higher occurrence in non-white adolescents with T2D [49,50]. Despite initiated treatments, complications such as hypertension, dyslipidaemia, and microalbuminuria persisted post-diagnosis similar to other studies, underscoring the failure of current management in halting disease progression [51,52].

### 4.4. Treatment

Ninety-five percent of our cohort received metformin at diagnosis, in line with recommended practice; however, 18% required dose reduction, and 16% discontinued due to poor tolerance [27,40]. Basal and prandial insulin treatment rose to 73% and 38% of patients, respectively, after 3 years. Long-acting insulin dosage increased by 23%, while short-acting insulin decreased by 2% compared to diagnosis. The deterioration in HbA1c at the end of the first year may result from delayed insulin escalation, non-adherence, and efforts to minimise doses alongside diminishing beta-cell function. Only 24% (*n* = 9/37) of patients sustained the HbA1c target < 48 mmol/mol for over a year. Therapies known to be effective in adults may not yield the same results in paediatric patients. This is best demonstrated with respect to glycaemic failure rates on metformin monotherapy, which in adults can range from 21 to 42%, whilst in youths, the rates are much higher as reported in the TODAY trial and UKPDS study [26,53]. There is an urgent need for alternative drugs other than insulin and metformin for paediatric T2D, with liraglutide’s recent licensing and new NICE guidance for use of GLP1-RAs and SGTL2 inhibitors in paediatric T2D marking a positive step forward [54].

Our study’s strengths include an extended duration enabling three years of follow-up for most participants, and the Royal London Hospital’s status as a high-volume diabetes centre facilitating a larger single-centre cohort compared to national averages. Utilising Twinkle, a specialised electronic database for paediatric diabetes patients, ensured robust patient identification and data collection. However, there were also limitations in our study. This included the retrospective nature of the analysis, introducing detection bias and missing data, which limit the temporal causality assessment between glycaemic control and complications. The relatively small sample size and regional focus in East London restrict the generalisability of our results. At the time of diagnosis for our patients, no other medications were licensed apart from metformin and insulin. Uncollected mental health and complication treatment data further constrain insights. Additionally, there may be patients managed in primary care or by adult diabetologists that were not captured in our cohort.

## 5. Conclusions

In conclusion, our study has highlighted the increasing incidence of youth-onset T2D, the severity of the disease at diagnosis with a high burden of co-morbidities and complications, and the ineffectiveness of current management strategies in achieving adequate clinical outcomes. Recently, guidelines for the management of paediatric T2D have been developed by the Association of Children’s Diabetes Clinicians (ACDC) in the UK. These guidelines, together with the increased awareness and the initiation of a National Working Group for Paediatric Type 2 Diabetes, may help to improve the outcomes for these patients. T2D in this age group has a complicated social and environmental context influencing its progression, and it is clear that management should evolve to a multifaceted and holistic approach. Future research should investigate the reasons behind the high therapeutic failure rates and the predictive factors for early complication development. Future management approaches must address the challenges related to adherence, barriers to behavioural changes, routine complication assessments, and enhancement of clinical support and follow-up, particularly during the transition to adult services.

## Figures and Tables

**Table 1:** Baseline characteristics at diagnosis.

Characteristics	Frequency
Total number of patients, *n*	40
Age at diagnosis in years, mean (SD)	13.9 (1.73)
Minimum	9.5
Maximum	17.6
Females age	13.7 (1.89)
Males age	14.3 (1.41)
Gender, *n* (%)	
Female	25 (63%)
Male	15 (38%)
Ethnicity, *n* (%)	
Bangladeshi	22 (55%)
Indian	0 (0%)
Pakistani	2 (5%)
African	3 (8%)
Caribbean	2 (5%)
White	3 (8%)
Other	6 (15%)
Non-disclosed	2 (5%)
Co-morbidities, *n* (%)	
Yes	27 (68%)
No	10 (25%)
Unknown	3 (8%)
Positive Autoantibodies, *n* (%)	2 (5%)
Baseline Clinical Parameters, mean (SD)	
BMI (*n* = 38)	32.4 (6.71)
BMI SDS (*n* = 38)	2.87 (0.70)
HbA1c (*n* = 27)	75.2 (21.0)
Random blood glucose (*n* = 18)	10.6 (4.14)
Duration of follow-up in months, mean (SD)	26.6 (16.3)
Age at transition to adult care in years, mean (SD)	17.7 (1.35)

**Table 2:** Sub-analysis of clinical parameters by sex.

Clinical Parameters	Number of Patients (n)	Female	Number of Patients (n)	Male	*p* Value
Age in years, mean (SD)	25	13.7 (1.89)	15	14.3 (1.41)	0.359
BMI in kg/m^2^, mean (SD)					
At diagnosis	25	31.7 (5.73)	13	33.8 (8.35)	0.352
At 12 months	17	31.9 (4.74)	10	34.7 (8.49)	0.651
At 24 months	16	32.2 (5.27)	9	34.1 (8.79)	0.910
BMI SDS, mean (SD)					
At diagnosis	24	2.79 (0.75)	13	3.02 (0.60)	0.340
At 12 months	15	2.84 (0.66)	10	3.02 (0.61)	0.493
At 24 months	14	2.81 (0.76)	9	2.90 (0.77)	0.794
HbA1c in mmol/mol, mean (SD)					
At diagnosis	19	77.4 (22.02)	8	69.9 (18.37)	0.403
At 12 months	16	60.9 (21.92)	9	66.7 (31.99)	0.977
At 24 months	15	65.8 (24.38)	8	69.4 (35.41)	0.897

**Table 3:** Sub-analysis of clinical parameters by time period of diagnosis 2008–2013 versus 2014–2018.

Clinical Parameters at Diagnosis	Number of Patients (n)	2008–2013	Number of Patients (n)	2014–2018	*p* Value
Age in years, mean (SD)	13	14.3 (1.62)	27	13.7 (1.78)	0.314
BMI in kg/m^2^, mean (SD)	13	30.2 (5.95)	25	33.6 (6.90)	0.136
BMI SDS, mean (SD)	13	2.52 (0.81)	24	3.06 (0.56)	0.024 *
HbA1c in mmol/mol, mean (SD)	8	89.0 (19.20)	19	69.4 (19.24)	0.023 *

* *p* < 0.05.

**Table 4:** Frequency of complications at diagnosis and during follow-up.

Complications	Number of Patients/Number of Patients Assessed at Diagnosis, *n* (%)	Number of Additional Patients/Number of Patients Assessed during Follow-Up, *n* (%)
Hypertension (BP > 95th centile)	9/21 (43%)	13/37 (35%)
Raised ALT	6/30 (20%)	8/30 (27%)
Microalbuminuria (UACR > 3 mg/mmol)	2/14 (14%)	12/36 (33%)
Sleep apnoea	6/26 (23%)	0/26 (0%)
Fatty liver (on ultrasound)	7/24 (29%)	6/21 (19%)
Abnormal lipid profile		
High Cholesterol (>5 mmol/L)	9/30 (30%)	10/36 (28%)
High LDL (>2.6 mmol/L)	13/26 (50%)	19/36 (53%)
High Triglycerides (>1.7 mmol/L)	14/29 (48%)	13/32 (41%)
Low HDL (<0.9 mmol/L)	9/29 (31%)	10/32 (31%)

**Table 5:** Sub-analysis of HbA1c for sex and time period of diagnosis.

HbA1c in mmol/mol, Mean ± SD	Females (n)	Males (n)	*p* Value	2008–2013 (n)	2014–2018 (n)	*p* Value
At diagnosis	77.4 ± 22.0 (19)	69.9 ± 18.4 (8)	0.403	89.0 ± 19.2 (8)	69.4 ± 19.2 (19)	0.023 *
3 months	56.0 ± 16.0 (19)	52.6 ± 21.3 (8)	0.659	63.0 ± 21.7 (10)	50.2 ± 12.8 (17)	0.064
6 months	52.3 ± 15.4 (16)	69.3 ± 47.0 (6)	0.201	58.7 ± 16.8 (6)	56.3 ± 31.0 (16)	0.859
12 months	60.9 ± 21.9 (16)	66.7 ± 32.0 (9)	0.977	69.3 ± 29.6 (9)	59.4 ± 23.1 (16)	0.362
24 months	65.8 ± 24.4 (15)	69.4 ± 35.4 (8)	0.897	67.8 ± 28.9 (10)	66.5 ± 28.3 (13)	0.912

* *p* < 0.05.

## Data Availability

The data presented in this study are available on request from the corresponding author. The data are not publicly available due to patient confidentiality.

## Supplementary Materials

The following supporting information can be downloaded at: https://www.mdpi.com/article/10.3390/children11020173/s1, Table S1: Treatment regimen for patients at diagnosis and during follow-up; Figure S1: BMI SDS for individual patients at diagnosis and during the first 24 months after diagnosis; Figure S2: Mean doses of prescribed insulin during follow-up; Figure S3: HbA1c in individual patients during transition to adult services.
