# Supplementary material for: Exploring the Surge in Paediatric Type 2 Diabetes in an Inner-City London Centre—A Decade-Long Analysis of Incidence, Outcomes, and Transition
Source: Children (Basel). 2024 Jan 29;11(2):173. doi: 10.3390/children11020173 (PMC10887333; doi:10.3390/children11020173)

Supplementary Materials

Table S1. Treatment regimen for patients at diagnosis and during follow-up. The majority of patients were initiated on metformin at diagnosis, with around a third of patients being started on long-acting and short-acting insulin. Mean doses of insulin did not change significantly from diagnosis to 36 months follow-up.

Clinical Parameters	Number of patients, n (%)	Mean dose (SD) (mg/d or U/kg)
Metformin		
At diagnosis	38/40 (95%)	894.7 (370.5)
Stopped metformin during follow-up	6/38 (16%)	-
Reduced dose during follow-up	7/38 (18%)	-
Max. dose during treatment	38/40 (95%)	1921.1 (273.3)
Long-acting insulin		
At diagnosis	14/37 (38%)	0.30 (0.16)
At 12 months	17/21 (81%)	0.29 (0.20)
At 24 months	12/17 (71%)	0.38 (0.22)
At 36 months	11/15 (73%)	0.37 (0.17)
Short-acting insulin		
At diagnosis	6/18 (33%)	0.42 (0.20)
At 12 months	7/15 (47%)	0.44 (0.27)
At 24 months	8/16 (50%)	0.38 (0.21)
At 36 months	5/13 (38%)	0.41 (0.22)
Total dose insulin		
At diagnosis	14/25 (56%)	0.48 (0.37)
At 12 months	15/18 (83%)	0.44 (0.38)
At 24 months	11/14 (79%)	0.65 (0.42)
At 36 months	10/12 (83%)	0.56 (0.39)

Figure S1. BMI SDS for individual patients at diagnosis and during the first 24 months after diagnosis. All patients with BMI SDS at diagnosis and one other time point in the 24 months are shown (n=30). Each line represents an individual patient. Dotted lines represent females; solid lines represent males. Red lines represent patients diagnosed in 2008–2013 and blue lines represent patients diagnosed in 2014–2018.

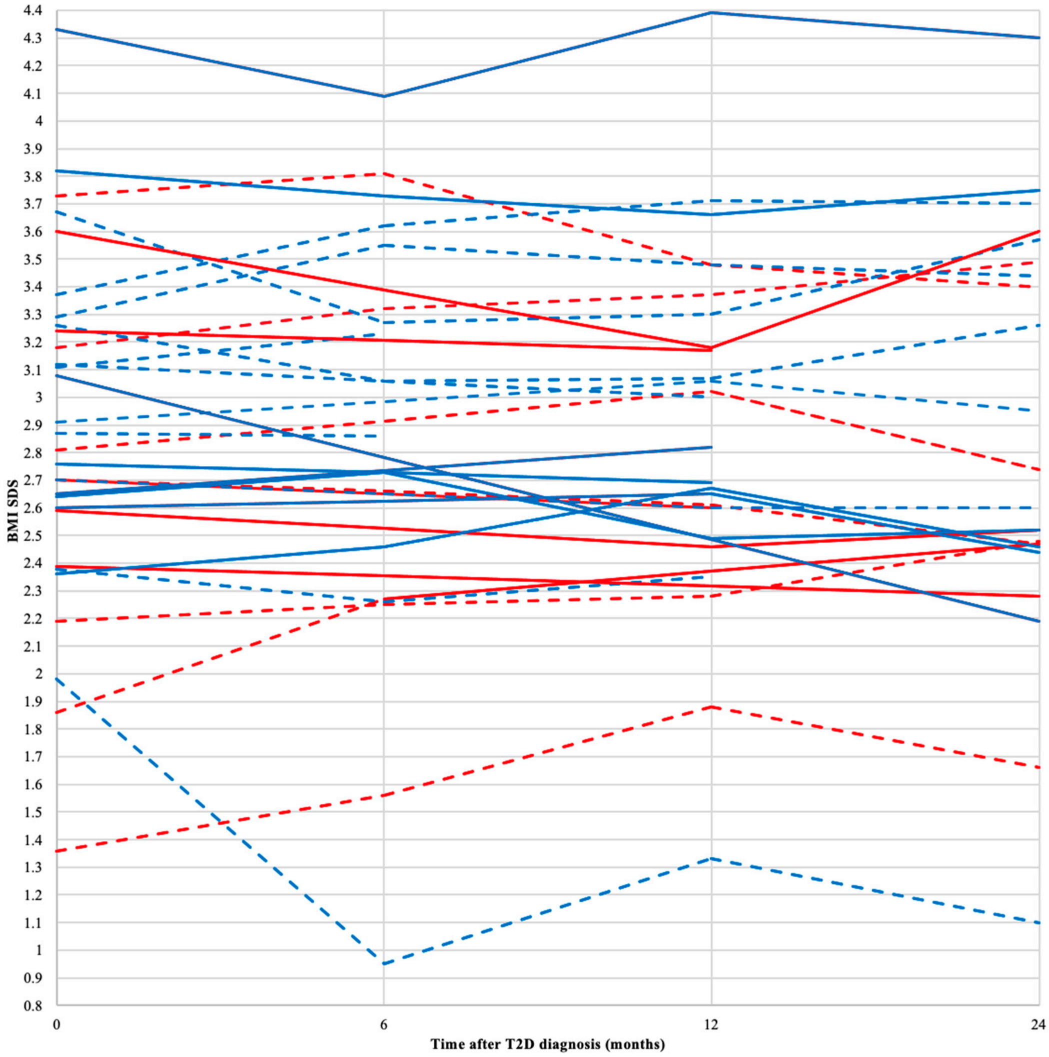

Figure S2. Mean doses of prescribed insulin during follow-up. Mean doses of long- and short-acting and total dose of insulin were collected at diagnosis, first year post-diagnosis, and second year post-diagnosis and expressed as U/kg. Mean and SD are shown and number of patients with available insulin doses at each time point (n). Mean doses of long- and short-acting insulin and total insulin did not change after diagnosis during the three years of follow-up.

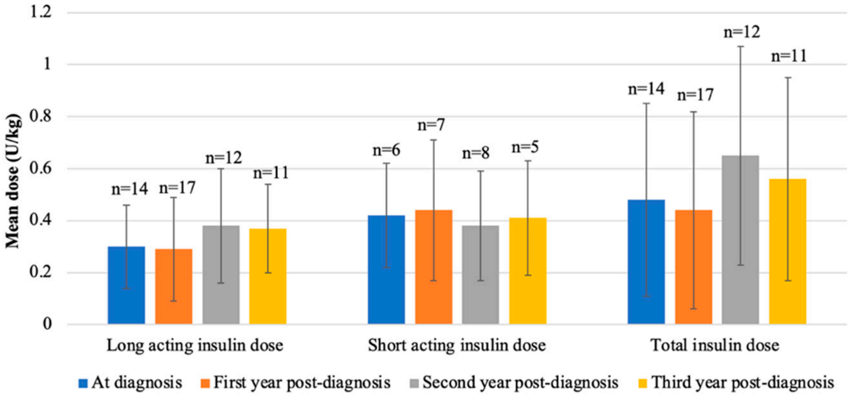

Figure S3. HbA1c in individual patients during transition to adult services. HbA1c was available for 9 patients in our cohort that transitioned from 2017. HbA1c for these patients are shown at diagnosis, at transition, and at the first and second year after transition. The year of transition is indicated for each individual patient (*). Patients 2 and 5 had no available HbA1c data at the second year after transition. Patients 4 and 9 were in remission at transition, but whilst patient 9 continued to sustain this remission, patient 4 relapsed by the second year post-transition.

**Transition year for each patient*

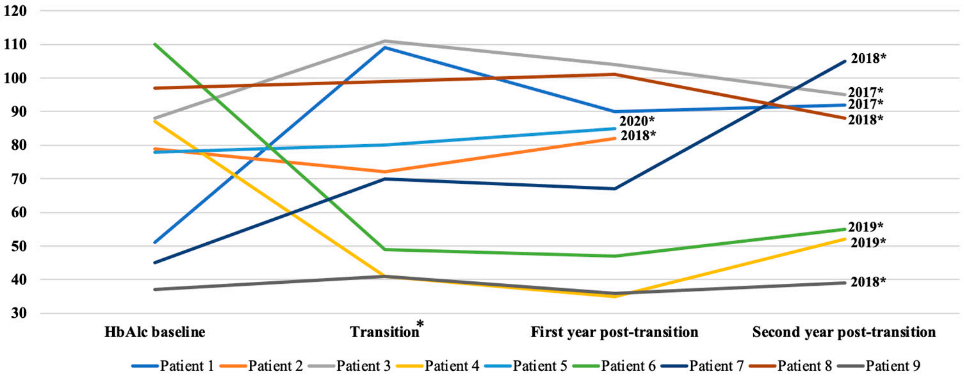

Supplement: Supplementary file 1 [file children-11-00173-s001.zip › children-2801815-supplementary.pdf]
